# A novel cost function to estimate parameters of oscillatory biochemical systems

**DOI:** 10.1186/1687-4153-2012-3

**Published:** 2012-05-16

**Authors:** Seyedbehzad Nabavi, Cranos M Williams

**Affiliations:** 1 Department of Electrical and Computer Engineering, North Carolina State University, Raleigh, NC, USA

## Abstract

Oscillatory pathways are among the most important classes of biochemical systems with examples ranging from circadian rhythms and cell cycle maintenance. Mathematical modeling of these highly interconnected biochemical networks is needed to meet numerous objectives such as investigating, predicting and controlling the dynamics of these systems. Identifying the kinetic rate parameters is essential for fully modeling these and other biological processes. These kinetic parameters, however, are not usually available from measurements and most of them have to be estimated by parameter fitting techniques. One of the issues with estimating kinetic parameters in oscillatory systems is the irregularities in the least square (LS) cost function surface used to estimate these parameters, which is caused by the periodicity of the measurements. These irregularities result in numerous local minima, which limit the performance of even some of the most robust global optimization algorithms. We proposed a parameter estimation framework to address these issues that integrates temporal information with periodic information embedded in the measurements used to estimate these parameters. This periodic information is used to build a proposed cost function with better surface properties leading to fewer local minima and better performance of global optimization algorithms. We verified for three oscillatory biochemical systems that our proposed cost function results in an increased ability to estimate accurate kinetic parameters as compared to the traditional LS cost function. We combine this cost function with an improved noise removal approach that leverages periodic characteristics embedded in the measurements to effectively reduce noise. The results provide strong evidence on the efficacy of this noise removal approach over the previous commonly used wavelet hard-thresholding noise removal methods. This proposed optimization framework results in more accurate kinetic parameters that will eventually lead to biochemical models that are more precise, predictable, and controllable.

## 1 Introduction

Oscillatory biochemical pathways are an important class of biochemical systems [1,2] that play significant roles in living systems. For instance, "circadian rhythms" are fundamental daily time-keeping mechanisms in a wide range of species from unicellular organisms to complex eukaryotes [3]. One of their most important roles is in regulating physiological processes such as the sleep-wake cycle in mammals [4]. "Cell cycles" are also another vital class of biochemical oscillations. The cell cycle is the sequence of events by which a growing cell replicates all its components and divides into two daughter cells [5]. Inappropriate cell proliferation due to malfunctioning cell cycle control mechanisms can cause development of certain types of cancers [5]. There are also other classes of biochemical rhythms such as cardiac rhythms [6], ovarian cycles [7] and cAMP oscillations [8] that have their own significance in systems biology.

A complete modeling of a biochemical system includes characterization of all nonlinear structures of the network along with the associated kinetic rates. In other words, without fully identifying all the kinetic parameter values, these models are still incomplete even if the full structure of the model has been determined. Few kinetic rates are available directly from experimentation or literature. Most of them, however, have to be estimated by parameter fitting techniques to complete the modeling of the biochemical pathway. Thus, a mathematical framework is needed to fit the kinetic parameters using the observables. Optimization frameworks that focus specifically on estimating parameters associated with biochemical pathways have received much attention in recent years [9-14].

Two main issues in estimating kinetic parameters in biochemical systems are data related issues and computational issues [14]. The measurement dataset used to fit these parameters are usually noisy and incomplete. Measurement datasets are also affected by uncertainties related to experimental conditions such as temperature and light [14]. Much study is done recently to reduce noise for different biochemical signals [15-17]. Mostacci et al. [15] proposed a denoising method for mass spectrometry data by integrating wavelet soft thresholding and principal component analysis. Weng et al. [16] suggested a noise removal approach for oscillatory ECG signals based on a recently developed method known as empirical mode decomposition. Ren et al. [17] also developed a method of denoising biochemical spectra by introducing a new thresholding function integrated with the "translation invariant" approach to lower the root mean square error (RMSE) in the measurements in comparison to the traditional soft and hard thresholding methods.

The computational issues include the challenges optimization algorithms face when identifying an optimal fit to measurement data. There are problems with optimization methods such as slow convergence toward global optima, complicated error surfaces and lack of convergence proofs [14]. Much study has been done to address these issues in parameter estimation in biochemical systems [12,13,18-21]. Zhan et al. proposed a method to reduce the computational time of each trial by integrating the spline functions theory with nonlinear programming to eliminate the need of solving the system of ordinary differential equations (ODEs) [21]. Rodriguez-Fernandez et al. [12] suggested a hybrid optimization method to speed up the convergence toward the global optima. A variety of different algorithms has also been adapted to perform the inverse problem. A comprehensive list of such studies is provided in [14].

Furthermore, heuristic approaches have been developed to address the optimization problem in fitting parameters in oscillatory systems [9-11]. These methods improved the optimization by constructing error functions based on the features extracted from the data. Locke et al. [11] proposed a cost function based on the comparison of entrained period, phase and strength of oscillation for the circadian clock in *Arabidopsis thaliana*. Also, Zeilinger et al. [10] performed another parameter estimation approach for the *A. thaliana *model by investigating amplitudes of some species in dark/light cycles, periods under dark and light conditions and the period of one mutant phenotype under constant light. In [9], Bagheri et al. built up an optimization process to model *Drosophila melanogaster *circadian clock by defining three cost functions based on free running period, light/dark entrained period, differences in amplitude and differences in the phase of the components in the system. These methods are more applicable for problems where characteristics in the system and/or data can be exploited to improve the performance of the parameter estimation. These methods, however, require more information about the system than purely data-driven comparison methods. For instance, the cost function proposed in [9] needs the period information of both the light and dark cycles of their investigated model, which requires a greater level of first principles knowledge. These methods are also model specific, which makes it difficult to apply them to general oscillatory systems. For example, the dark/light cycle characteristics that were introduced in parameter fitting problem of [10] may not be a suitable feature for parameter fitting of non-circadian biorhythms.

This article focuses on the problem of estimating the kinetic parameters in oscillatory biochemical systems. We show that periodicity in the measurements of oscillatory systems results in irregularly surface properties of the LS cost function leading to numerous local minima. These multiple local optima cause premature convergence of even robust optimization algorithms. This eventually results in incorrect estimates, bad predictions of dynamics, and incorrect acceptance of functional hypotheses. This, compounded with uncertainties or noisy measurements leads to a difficult estimation problem to solve.

We develop a parameter estimation framework to address these issues by integrating information of oscillatory systems in the modeling process (parameter estimation and denoising). This periodic information is used to build a cost function with better surface properties. Our proposed cost function takes advantage of the basic properties of these oscillatory systems, which allows us to generalize our cost function to a variety of biochemical systems with sustained oscillations. The proposed cost function also needs less first principles knowledge to generate the cost function in comparison to the previous methods that was developed for oscillatory systems [9-11]. We verified for three oscillatory biochemical systems that our proposed cost function results in increased ability to estimate accurate kinetic parameters as compared to the traditional LS cost function. We combined this cost 6 function with an improved denoising method that also leverages periodic characteristics embedded in the measurements to effectively reduce noise. The results provide strong evidence on the efficacy of this noise removal approach over the previous commonly used wavelet hard-thresholding noise removal method. This proposed optimization framework results in more accurate kinetic parameters that will eventually lead to biochemical models that are more accurate, predictable, and controllable.

## 2 Methodology

This study considers deterministic, nonlinear oscillatory biochemical pathways described by ODEs as shown in (1):

(1) $$\begin{array}{ccc} \dot{x}\left(t\right) & =f\left(x\left(t\right),p\right) & t_{0}<t<t_{e},\\ x_{0} & =x\left(t_{0}\right). \end{array}$$

Here, **x **∈ ℝ^*m*×1 ^is the state vector of the *m *components of the pathway, **p **∈ ℝ^*n*×1 ^is the vector of *n *kinetic parameters, **f: **ℝ^*m*×1 ^→ ℝ^*m*×1 ^is a nonlinear vector function, **x**_0 _∈ ℝ^*m*×1 ^is the vector of the initial component concentrations at time *t*_0 _and *t*_0 _*< t < t_e _*represents the time of interest.

Optimization describes the approach of estimating the kinetic parameters (**p**) of the system described in (1) that cannot be measured directly using a set of experimental data. The criteria for verifying the quality of the estimates is often an error function such as Φ as shown in (2). This function quantifies the ability of the estimates to reproduce the same results as the measurements. This objective function is minimized such that $p=\hat{p}$results in the minimum value of Φ. In that way, $\hat{p}$ is called the estimated point.

(2) $$\hat{p}=\text{arg }\underset{p}{\text{min }}\Phi \left(p\right)$$

One of the most common cost functions is the *least square (LS) estimator *[22]. This estimator is based on the sum of the squares of the point by point errors between measured experimental data and the simulated measurements from the estimated model as described in (3):

(3) $$\Phi \left(p\right)=\overset{N_{x}}{\underset{i=1}{\sum }}\overset{N_{m}}{\underset{j=1}{\sum }}{\left(x_{ij}-{\hat{x}}_{ij}\left(p\right)\right)}^{2}.$$

Here, *x_ij_*, is the measurement at time *j *of the *i*th state of the system, ${\hat{x}}_{ij}$ is the reproduced data at time *j *for the *i*th state of the system given some parameter **p**, *N_m _*is the number of time points where measurements are obtained and *N_x _*is the number of measured outputs (in this manuscript, they are considered to be the measured states of the system).

**Figure 1:**
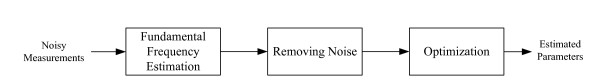
**The implemented process of parameter estimation for oscillatory biochemical systems**.

The objective of this article is to propose a method to estimate the kinetic parameters for a given oscillatory biochemical system of the form (1) using the noisy measurements of the system states. We first captured periodic information of the measurements. This information is used to improve noise reduction and generate an error cost function with better optimization properties. The next step implements a modified wavelet hard thresholding denoising approach that uses the previously obtained periodic information of the measurements to further reduce uncertainties in noisy data. We then generate our proposed cost function by integrating the periodic information obtained in the first step with the simulated data and measurements. We searched the surface of the proposed cost function with a series of optimizers in a hybrid manner. Hybrid methods use global optimization followed by local 8 [24]. We used a frequency-based method called optimization [12,23]. Global and local optimization algorithms were used in succession to further improve optimization results. A block diagram of our approach for parameter estimation of oscillatory systems is shown in Figure 1. The following sections outline each of the blocks of this diagram.

### 2.1 Fundamental frequency estimation

The fundamental frequency is an essential metric for assessing the underlying oscillatory characteristics in a signal and is a critical step in developing our proposed cost function and noise removal method. The fundamental frequency is the oscillation frequency of the continuous data. The measurements are samples of this continuous-time signal. If one assumes a periodic waveform of *x*(*t*) such that:

(4) x(t)=x(t+kT)∀k∈ℤ,

the smallest value of *T *≠ 0 for which (4) is valid is the *"fundamental period" *of oscillation. The inverse of the fundamental period is the fundamental frequency (*f*_0_). Several approaches has been proposed to estimate *f*_0 _[24]. We used a frequency-based method called *component frequency ratio *[24] to extract the fundamental frequency of the measured data due to the fact that the time-series methods may not be adequate for biochemical measurements due to their low rate of sampling and low temporal resolution. This method starts with transforming the data to the Fourier domain by taking their Fourier transform. The locations of the peaks in the spectrum are then identified. The peaks in the frequency spectrum are the harmonics of the fundamental frequency. The final step is to find the greatest common factor of these frequencies in which peaks occur.

#### 2.1.1 Effect of noise on estimation of *f*_0_

**Figure 2:**
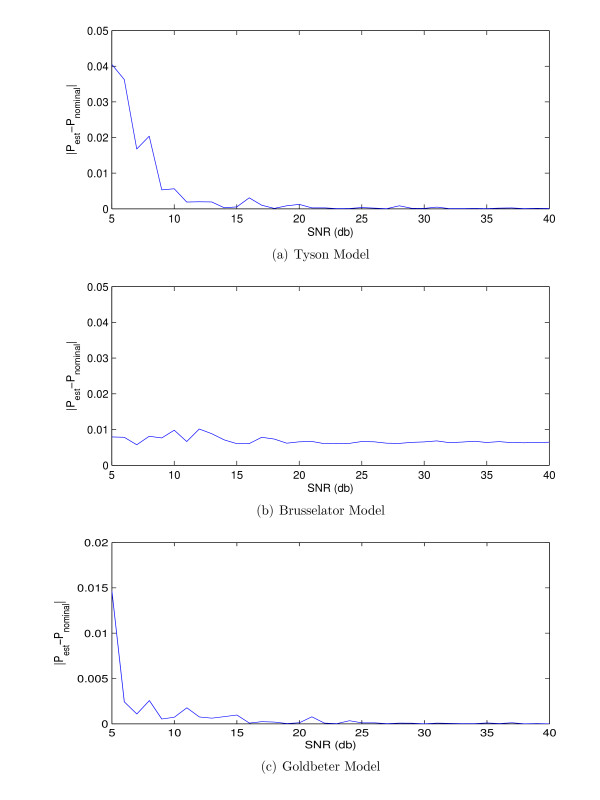
**The error in estimating of the fundamental period versus the amount of noise in measurements for (a) Tyson model **[25], **(b) Brusselator model **[26], **(c) Goldbeter model **[27].

This section investigates the effect of noise on estimation of *f*_0_. We considered three model systems identified from the literature: the two-state Tyson model [25], the two-state Brusselator model [26] and the five-state Golbeter model [27]. We considered the measurements of the states of these models with the sampling rate equal to 1 (sample/hour). Then, we added AWGN noise with various SNRs to these signals and we estimate their fundamental period using the method *component frequency ratio*. Figure 2 shows the absolute error between the estimated and the nominal fundamental period of the three models for various amount of additive noise.

Figure 2 shows that the method used to estimate the fundamental period is robust enough to the additive noise.

### 2.2 Removing noise

**Figure 3:**
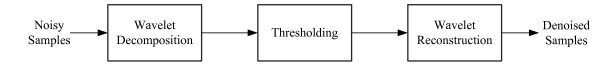
**The commonly used thresholding algorithm to remove noise**.

One common approach to reduce noise in measurements is wavelet hardthresholding [28], which employs a thresholding function over the wavelet coefficients of the noisy data samples. The motivation of using wavelets is that it provides an appropriate basis to separate noise from signal in the wavelet domain. The small wavelet coefficients are more likely to be noise and large coefficients are more likely to be components of the original signal. Thus, noise could be eliminated approximately from the signal by thresholding the wavelet coefficients [29]. The steps of the noise removal procedure using this method are shown in Figure 3:

#### 2.2.1 Improving the hard-thresholding method in oscillatory systems

**Figure 4:**
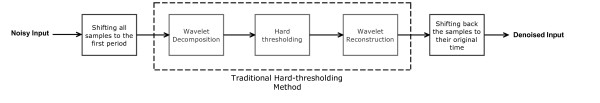
**The proposed noise removal steps**.

Samples of oscillatory signals contain repetitive patterns if they are taken over multiple periods. Thus, we hypothesized that is possible to take advantage of data oscillation to improve the denoising of the samples provided that their fundamental frequency is given or can be estimated. We modified the denoising procedure of oscillatory signals by adding two additional steps to the traditional hardthresholding method as depicted in Figure 4. Two assumptions have been made about the noisy oscillatory data. First, the fundamental period of the data is not an integer multiple of the sampling rate. Otherwise, it is not possible to increase the resolution of the data by shifting them in this method. Second, we assumed to have the measurements of more than one period of the data. Otherwise, there will be no way to estimate the fundamental period of the measurements.

The first step in Figure 4 is shifting all samples to the first period of the data. This is based on the following steps:

1. Partition the measurements *X*(*nT_s_*) based on their calculated fundamental period to the sets of *X_k _*according to (5)

(5) $$X_{k}\left(nT_{s}\right)\;=X\left(nT_{s}\right)\;kT\leq nT_{s}<\left(k+\text{ 1}\right)T,\;\;0\leq k<\;m,$$

where *T_s _*is the sampling period and T  is the fundamental period of the measurements *X*.

2. Shift each *x_k _*by the value of kT. This will result in a single period of the measurements with higher resolution. The shifted versions of *x_k_*'s are calculated based on (6)

(6) $$\overset{N-1}{\underset{k=0}{⋃}}x_{k}\left(nt-kT\right)$$

**Figure 5:**
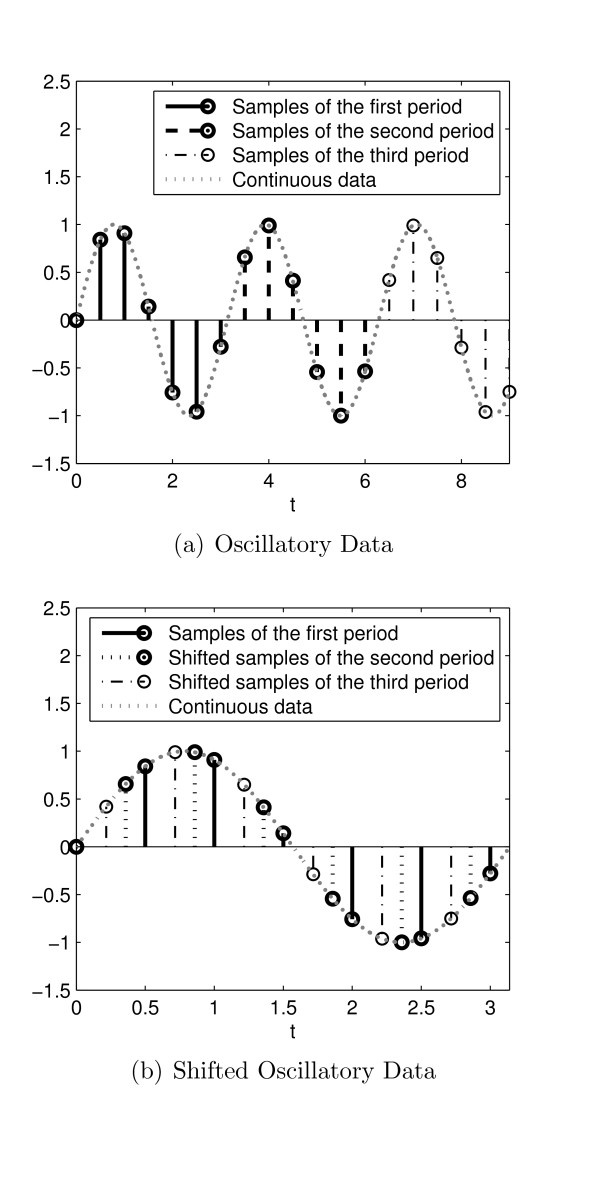
**The samples of three periods of the function x(t) = sin 2*π*t with the rate of 2 (sample/sec) and their shifted version based on the procedure outlined in (5) and (6)**.

Figure 5 illustrates the samples of a sine function of *x*(*t*) = sin 2*πt *with the rate of 2 (sample/sec) and its shifted version. We see that Figure 5b shows only the first period of the sine function but with higher resolution.

**Figure 6:**
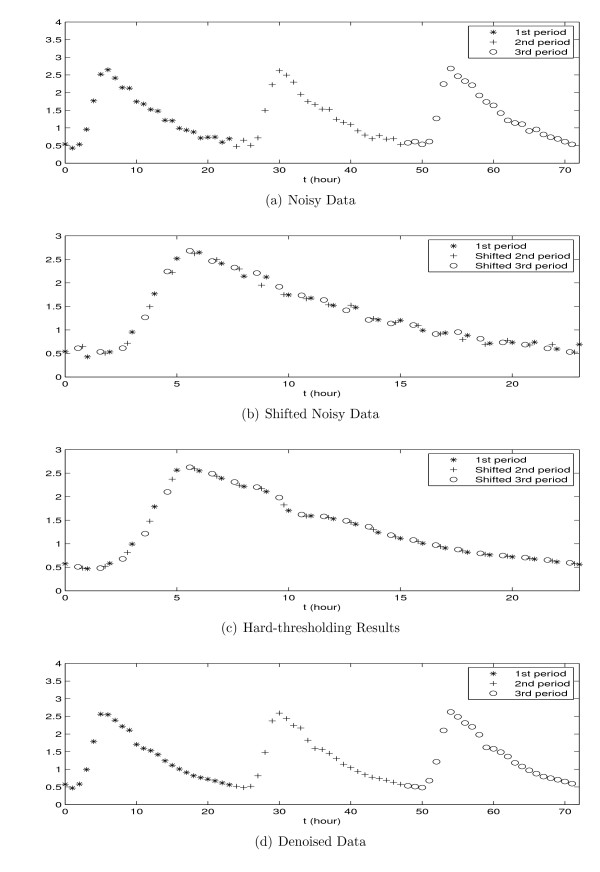
**The proposed noise removal steps (a) the original noisy measurements, (b) shifted version of the noisy data based on fundamental period, (c) the thresholding results over shifted version of the data, (d) moving back all samples to their original time**.

Figure 6 shows the denoising process for the *in-silico *measurements of the [*M*] component with sampling rate = 1 (sample/hour) in the model of the circadian clock in *D. melanogaster *proposed by Tyson [25]. The noise in the measurements is additive white Gaussian noise (AWGN) with SNR = 20 dB. Figure 6b shows the shifted version of the noisy measurements of Figure 6a using a calculated fundamental period of 24.21.

Wavelet decomposition, thresholding and reconstruction are then applied to this "shifted version" of the noisy data. MATLAB was used to implement a three level wavelet decomposition using the "*Daubechies 6" *wavelet and the threshold value equaling 0.3. The wavelet type, number of levels, and the threshold value were chosen empirically and may vary from system to system. The results are shown in Figure 6c. The final step is to reconstruct the original signal by shifting the samples back to their respective periods (Figure 6d).

**Figure 7:**
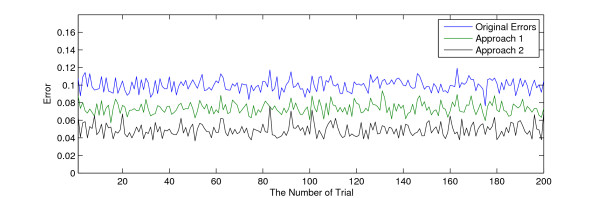
**Comparison of the two noise removing methods for an oscillatory biochemical dataset ($[P_t]$ in the Tyson model)**. Original errors: sum of the squared errors between noisy data and the original data, Approach 1: sum of the squared errors between the original data and the denoised data using the traditional thresholding method, Approach 2: sum of the squared errors between the original data and the denoised data using the proposed noise removing method.

We compared the performance of the proposed denoising method and the traditional wavelet hardthresholding by taking the samples of the [*Pt*] component with sampling rate = 1 (sample/hour) in the Tyson model of circadian clock in *D. Melanogaster *[25]. Then, we added AWGN noise with SNR = 20 to the dataset in 200 trials. We then removed noise using two approaches: the traditional wavelet hardthresholding method [29] and our proposed 12 method. Figure 7 compares three errors for each of the 200 trials. (1) The RMSE between the noisy data and the original dataset (the original error), (2) the RMSE between the denoised data resulting from the traditional thresholding method and the original dataset (Approach 1), and (3) the RMSE between the denoised data resulting from the proposed denoising method and the original dataset (Approach 2). This figure shows that our proposed method of denoising is more effective at removing noise than the wavelet hardthresholding method, consistently lowering the RMSE between the original signal and the denoised signal.

#### 2.2.2 The effect of error in estimating *f*_0 _on proposed denoising method

**Figure 8:**
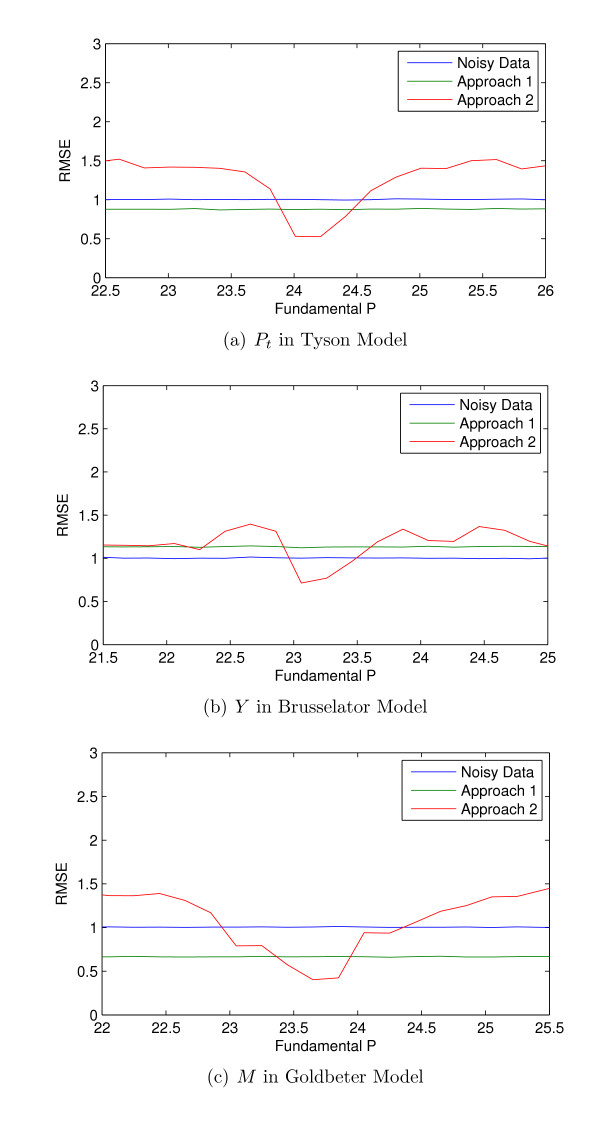
**The comparison of the RMSEs of noisy signal, the traditional hardthresholding method, and the proposed denoising method for various estimations of the fundamental period for three models of (a) Tyson with fundamental period of 24.17, (b) Brusselator with the fundamental period of 23.06, (c) Goldbeter with fundamental period of 23.65**.

This section investigates the impact of the inaccuracies of the fundamental period estimate on the proposed denoising method. We considered the samples of the components of the Tyson [25], Brusselator [26], and Goldbeter model [27] with sampling rate = 1 (sample/hour) and AWGN noise with SNR = 20. Then, we denoised the data with using the traditional wavelet thresholding (Approach 1) and the proposed denoising method (Approach 2) assuming inaccurate estimated fundamental period. Figure 8 compares the RMSEs of the results of these two methods and the noisy data for ranges of inaccurate estimated fundamental periods.

Figure 8 shows that the results of the proposed denoising method has lower RMSEs than the traditional wavelet thresholding with small errors in the estimation fundamental period. However, if the fundamental period is estimated with errors approximately more than 0.25 for these models, the proposed method does not yield lower RMSEs. However, Figure 2 shows that the error in fundamental period estimation due to noise is much smaller than the order of error that is considered in Figure 8.

### 2.3 Optimization

#### 2.3.1 Forming cost function

**Figure 9:**
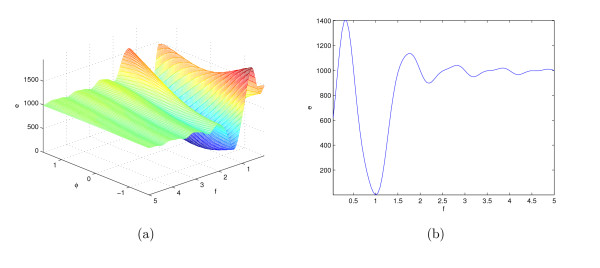
**The surface of LS cost function for the function shown in (7) versus the variation in parameters *ϕ *and *f *and its cross section for *ϕ *= 0**.

One big disadvantage of comparing point by point samples to build the LS cost function of (3) for oscillatory systems is the introduction of surface irregularities and numerous local optima. Let us consider a simple example of a sine function described in (7):

(7) y(n)=1+sin(2πfn/1000 +ϕ),

where *f *= 1 is the frequency and *ϕ *= 0 is the initial phase. Figure 9 illustrates the surface of the LS cost function (3) for ranges of the signal parameters, *f *and Φ.

Figure 9 shows significant rippling especially along the *f *direction of the LS cost function. This happens due to the varying degree of overlap between various periods of two oscillatory signals in the LS objective function along the *f *axis. This potentially results in numerous local basins of attractions that hinder the optimizer's ability to find the global optimum. These ripples are fundamental characteristics of the LS cost function for systems with oscillatory dynamics. This phenomenon can be observed for a large class of oscillatory systems especially along the parameter axes to which the fundamental frequency is more sensitive.

**Figure 10:**
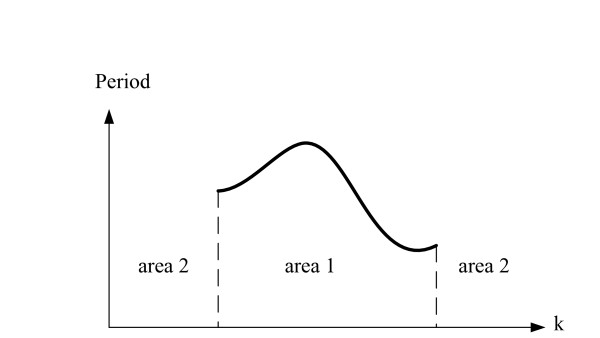
**Changes of the fundamental period of the sustained oscillation for ranges of values of a certain kinetic parameter for a hypothetical oscillatory system**.

Thus, we hypothesize that we can leverage information embedded in the data to produce a cost function with better surface properties, resulting in fewer local minima. This function is constructed in a piecewise manner based on the oscillatory characteristics of the simulated data at various parameter values. These characteristics are divided into two cases: sustained oscillations in the simulated data and no sustained oscillations in the simulated data. Sustained oscillation for a specific value of the parameter *k *is characterized by the fundamental period of the oscillations. A plot describing this is shown in Figure 10. All the parameter values for *k *in "area 1" produces sustained oscillations. This figure shows that the fundamental period of the sustained oscillation over this range may change. The values in "area 2", on the other hand, lead to dynamics that are not sustained oscillations.

If the simulated data are periodic, we introduced only the samples of one period of the data into the cost function. Likewise, only the samples of one period of the measurements will also be incorporated into this cost function. If the fundamental period of the measured data is not equal to the fundamental period of the simulated data, the signal with the smallest period is padded with zeros until the lengths of the signals are equal. This results in monotonic changes in error with respect to changes in fundamental period of the simulated data.

If the simulated data are nonperiodic as in area two of Figure 10, all time point measurements and the simulated data will be included in the cost function, resulting in the same cost function as the traditional LS objective function. Equation (8) describes the new proposed cost function for the ODE-based model of an oscillatory biochemical pathway (1).

(8) $$e\left(p\right)=\overset{N_{x}}{\underset{i=1}{\sum }}\overset{N_{z_{i}}}{\underset{j=1}{\sum }}{\left(z_{ij}-{\hat{z}}_{ij}\left(p\right)\right)}^{2},$$

where *z_ij _*and ${\hat{z}}_{ij}$ for periodic ${\hat{x}}_{i}$ are calculated as:

(9a) $$z_{ij}=\left\{\begin{array}{cc} x_{ij} & 0\leq t_{j}<T_{i}\\ 0 & T_{i}<t_{j}\leq \text{max}\left(T_{i},{\hat{T}}_{{}_{i}}\right) \end{array}\;,\right)$$

(9b) $${\hat{z}}_{ij}=\left\{\begin{array}{cc} {\hat{x}}_{ij} & 0\leq t_{j}\leq {\hat{T}}_{i}\\ 0 & {\hat{T}}_{i}<t_{j}\leq \text{max}\left(T_{i},{\hat{T}}_{i}\right) \end{array}\;.\right)$$

Otherwise, *z_ij _*and ${\hat{z}}_{ij}$ for non-periodic ${\hat{x}}_{i}$ are calculated as:

(10a) $$z_{ij}=x_{ij}$$

(10b) $${\hat{z}}_{ij}={\hat{x}}_{ij}.$$

Here, *x_ij _*is the measurement at time *t_j _*of the *i*th state of the system, ${\hat{x}}_{ij}$ is the simulated data at time *t_j _*for the *i*th state of the system. *z_ij _*and ${\hat{z}}_{ij}$ are the truncated and zero padded *x_ij _*and ${\hat{x}}_{ij}$, respectively, for the oscillatory ${\hat{x}}_{i}$. For non oscillatory ${\hat{x}}_{i}$, *z_ij_*, and ${\hat{z}}_{ij}$ are equal to *x_ij _*and ${\hat{x}}_{ij}$, respectively. $N_{z_{i}}$ is the length of the *z_i _*and ${\hat{z}}_{i}$. *N_x _*is the number of states of the system, *T_i _*is the fundamental period of the measurements (*x_i_*), which was computed using the component frequency ratio approach and ${\hat{T}}_{i}$ is the fundamental period of the simulated data $\left({\hat{x}}_{i}\right)$, which is estimated for each candidate parameter value. ${\hat{T}}_{i}$ was estimated using the YIN approach [30], which is a modified version of the time-domain autocorrelation method.

**Figure 11:**
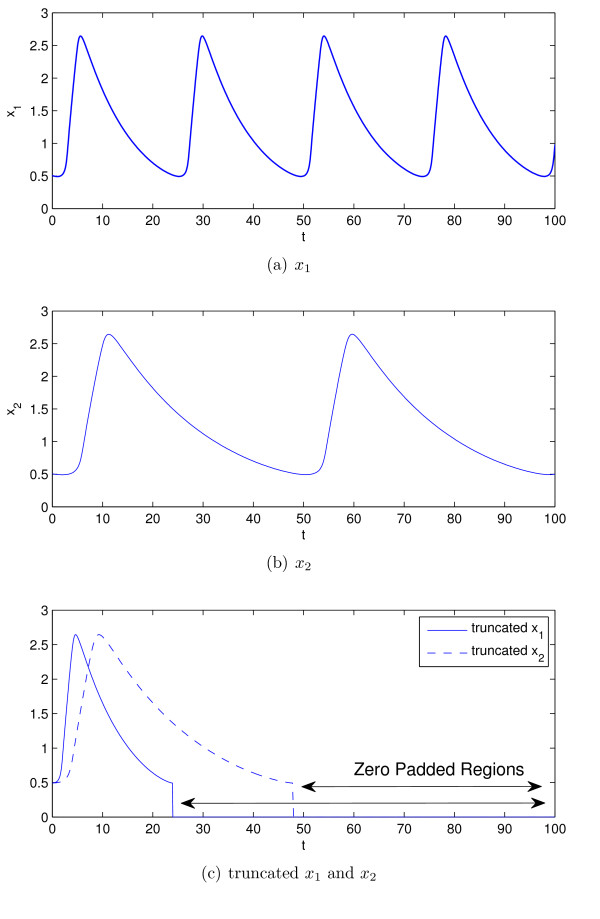
**Illustrating the two data sets with two different periods and the way the new cost function compares them; (a) the first data set with a period 24, (b) the second data set with period 48, (c) their truncated version to be compared by the cost function**.

Figure 11 illustrates how the proposed cost function compares two signals with different fundamental periods.

**Figure 12:**
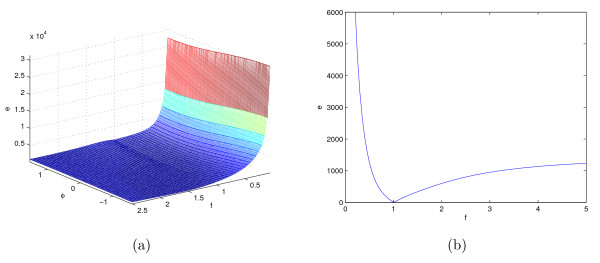
**The surface of the new cost function for the function shown in (7) versus changes in *ϕ *and *f *and its cross section for *ϕ *= 0**.

Figure 12 shows the surface of the proposed cost function of (8) for the sine function of (7). The global minimum of the proposed cost function also occurs at *f *= 1 and *ϕ *= 0 similar to the LS cost function of (3) shown in Figures 9. However, visual inspection of these two figures shows that the surface of proposed cost function is smoother than the surface of the LS cost function for the example of (7). We hypothesize that this improvement of the cost function surface will improve the performance of the optimization search algorithm.

##### The effect of error in estimating *f*_0 _on the performance of the cost function

The performance of the proposed cost function (8) is not affected significantly by errors in the estimation of the fundamental frequency of the measurements. This is because of the fact that the measurements used in (8) have a certain sampling rate. Basically, if the error of the estimated fundamental period is small with respect to this sampling rate, it will not affect the number of samples that lies in one fundamental period of the data. Also, adding or reducing one sample in the summation of (8) obviously will not change the performance of the proposed cost function dramatically.

#### 2.3.2 The optimization method

The optimization of the proposed cost function was performed using a hybrid approach. Hybrid methods, i.e. the combinations of global and local search methods, have been shown to yield results with smaller errors than global searches individually [12,23]. The global search algorithm that we adopt in this study is the "Genetic Algorithm", which is a widely-used approach of a class of global search methods called *evolutionary strategies *[31]. We used two consecutive local search methods of MATLAB [32] in this research. The first one was the derivative-based, constrained routine of fmincon, and the second one was the derivative-free routine of fminsearch that is based on the simplex algorithm [33].

## 3 Results

This section shows the results of the optimization process that was illustrated in Figures 1 using two cost functions: the LS cost function of (3) and our proposed cost function of (8). We used three model of Tyson [25], Brusselator [26], and Goldbeter [27]. We compare 15 independent runs of the optimization process for parameter estimation for each oscillatory model. We add AWGN noise with SNR = 20 to the data. We use our proposed noise removal method to remove noise. The surface of the two cost functions will be shown and compared for these three systems. Results at all the intermediate steps of the optimization will be presented for each of the 15 runs:

1. The global optimization (MATLAB ga routine).

2. The first local optimization (MATLAB fmincon routine)

3. The second local optimization (MATLAB fminsearch routine)

### 3.1 Comparison of two different cost function

The two cost functions of (3) and (8) are two different functions of the kinetic parameters which do not necessarily yield the same value for a given parameter set. Thus, a true comparison of the estimated parameters obtained from the two objective functions will require the LS score shown in (11) to equate the quality of the respective estimates. Equation (11) is basically the LS cost function summed only over the samples taken from the first fundamental period of the measurements. Introducing the measurements of only one period in computing the score creates a fair metric that shows the quality of estimated parameter sets.

(11) $$\text{score}\left(k\right)=\overset{Nx}{\underset{i=1}{\sum }}\overset{N_{T_{i}}}{\underset{j=1}{\sum }}{\left({\hat{x}}_{ij}\left(k\right)-x_{ij}\right)}^{2}.$$

Here, *N_x_, x_ij_*, and ${\hat{x}}_{ij}$ are defined as (3) and $N_{T_{i}}$ is the number of samples that are extracted in (0 *< t < T_i_*) assuming *T_i _*is the fundamental period of the *x_i_*.

### 3.2 Parameter estimation results for two-state Tyson model

The two-state Tyson model (BIOMD0000000036 in BioModels database [34]) is a mathematical model of the circadian clock in wild-type fruit flies, *D. melanogaster*. This organism has circadian clocks similar to mice and bread molds. This model, shown in (12), consists of two states and nine kinetic parameters. The nominal values of the parameters of this system are shown in Table 1.

(12a) $$\frac{dM}{dt}=\frac{v_{m}}{1+{\left(P_{t}\left(1-q\right)/2P_{crit}\right)}^{2}}-k_{m}M$$

(12b) $$\frac{dPt}{dt}=v_{p}M-\frac{k_{p1}P_{t}q+k_{p2}P_{t}}{J_{p}+P_{t}}-k_{p3}P_{t}$$

(12c) $$q=\frac{2}{\sqrt{1+8K_{eq}P_{t}}}$$

**Figure 13:**
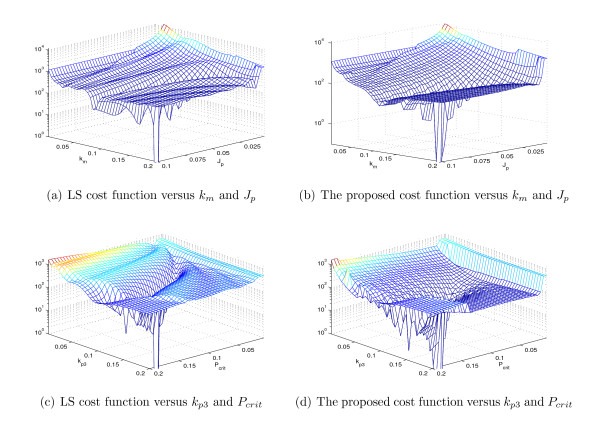
**The comparison of the surface of the two cost functions, left column: LS cost function, right column: the proposed cost function, for different values of *k_m_, J_p_, k*_*p*3 _and *P_crit _*in Tyson system, while changing two values of parameters, the rest of parameters are locked in their nominal value**.

Figure 13 shows the surfaces of the LS cost function and the proposed cost function of (8) for pairwise combinations of parameters *k_m _*and *J_p _*and *k*_*p*3 _and *P_crit _*over specific ranges. Characteristics of these parameters are representative of the characteristics of all kinetic parameters of the Tyson model (results are not shown). The values of the remaining parameters are held constant at their nominal values in all figures.

**Figure 14:**
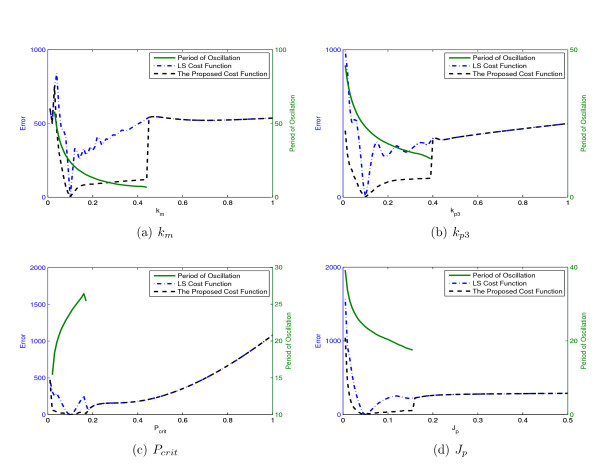
**The values of the LS cost functions, the proposed cost function and the fundamental period of the data for different values of 4 parameters of Tyson model *k_m_, k*_*p*3_, *P*_crit _and *J_p_***.

We see through visual inspection that our proposed cost function produces a smoother surface than that of the LS cost function for different values of the parameters *k_m_, k*_*p*3_, *P_crit_*, and *J_p_*. Figure 14 shows the cross-sections of the cost functions above (dashed lines) together with the fundamental period of the data (solid line) for ranges of values in the same order of magnitude as the nominal value.

Figure 14a shows that the system produces sustained oscillations only for *k_m _*in the range [0.03 0.44]. The fundamental period of the sustained oscillations falls from 58 to 6.6 along this range. This radical change in the fundamental period produces irregularities in the LS cost function over this interval. However, the proposed cost function maintains good surface properties in spite of this extreme change in the fundamental period of the system. This emphasizes that our proposed cost function addresses the issue of surface irregularities of the LS cost function caused by introducing multiple periods of the data in calculating the error. Figure 14b shows similar results.

Figure 14c, d shows that the fundamental period for different values of *P_crit _*is between 15.4 and 25.4 which is less than the changes in fundamental period that shown in Figure 14a, b. The LS cost function still shows varying levels of surface irregularities particularly along the *P_crit _*axis. The proposed cost function again shows smoother surface characteristics under these conditions as well.

#### 3.2.1 Results of parameter estimation

**Figure 15:**
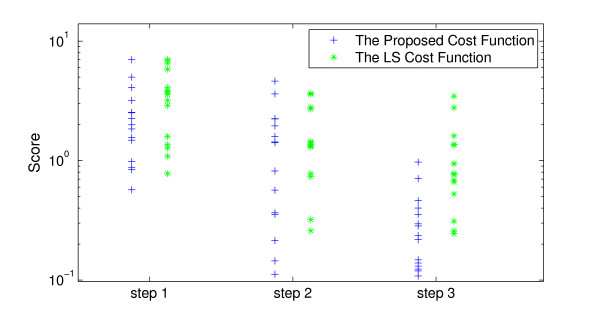
**The comparison of the computed scores resulted from the cost functions in 3 steps of optimization: Step 1: results of ga routine**. Step 2: results of fmincon routine. Step 3: results of fminsearch routine.

We assumed the measurements to be 100 samples of both [*M*] and [*P_t_*] components with the rate of one sample per hour and the AWGN noise of SNR = 20. We removed the noise using the proposed approach before the optimization step. The RMSE between the noisy samples and their real values of the samples were 0.0989. This was suppressed to 0.0413 after denoising. The population size was set to 200 and number of generations equals 50 for the ga routine. We calculated $N_{T_{i}}$ from (11) to be 24 for the Tyson model. The computed scores for the estimated parameters from the 15 runs of optimization are shown in Figure 15 at the three steps of the hybrid optimization process. The mean, median and the minima of the computed scores at each level for the two cost functions are also shown in Table 2. Figure 15 and Table 2 show visually and numerically that the optimization routine performs better using the proposed cost function than the LS cost function at all steps. These results are also consistent with our visual inspections of the cost functions in Figures 13 and 14.

**Table 1:** The results of optimization with minimum score for Tyson model.

Parameter	Nominal value	Estimation of the proposed cost function	Estimation of the LS cost function
*v_m _*	1	**1.1372**	0.9472
*k_m _*	0.1	0.1049	0.1097
*v_p _*	0.5	0.4668	0.4740
*k_p1 _*	10	**15.88**	**21.48**
*k_p2 _*	0.03	**0.0936**	**0.0927**
*k_p3 _*	0.1	**0.0766**	**0.0615**
*K_eq _*	200	**692.64**	**922.16**
*P_crit _*	0.1	0.1076	**0.1477**
*J_p _*	0.05	0.0511	**0.0738**
Score	0.1378	0.1084	0.2441

The values of kinetic parameters with minimum score derived from optimization using the proposed cost function and the LS cost function for the Tyson model. The bold values are the ones that are estimated incorrectly (error more than 10%). The score values are calculated based on (11)

**Table 2:** Statistics of optimization results for Tyson model.

	Step 1		Step 2		Step 3	
	
	Proposed	LS	Proposed	LS	Proposed	LS
Mean	2.4497	3.5838	1.4465	1.7760	0.3131	1.1116
Median	1.9998	3.5731	1.4117	1.3595	0.2354	0.7788
Min	0.5706	0.7788	0.1118	0.2589	0.1084	0.2441

Mean, median, and minimum of the score values shown in Figure 15

The optimized results with the lowest score out of 15 runs for the LS cost function and the proposed cost function are shown in Table 1.

The estimate results in the lowest score using noise-free measurements produces six of nine kinetic parameters with less than 10% errors (results not shown). Table 1 shows that the noisy case results in four of nine estimated parameters with more than 10% error. In both cases, proposed cost function yields more accurate results in comparison to the LS cost function. The large number of inaccuracies for the noisy case is more a result of system sloppiness versus inaccuracies of the estimation procedure [35,36], which results in 21 a wide range of parameters with similar system dynamics. It is evident that our proposed cost function was able to produce better overall system dynamics than the traditional LS cost function, which is clearly conveyed by the lower overall error. Our proposed method, similar to the LS cost function, only takes into account the accuracy of dynamics. Thus, the sloppiness can results in moderate level of parameter accuracy. Recently, Apgar et al. proposed an experiment design framework to improve estimates of sloppy parameters in biochemical models [37]. This, however, is beyond the scope of this article.

### 3.3 Parameter estimation for two-state Brusselator model

The Brusselator model was proposed by Prigogine for theoretical analysis of autocatalytic reactions [26]. This model consists of two states and four kinetic parameters as shown in (13). The nominal values of the parameters of this system are shown in Table 3.

(13a) $$\frac{dX}{dt}=k_{1}A+k_{2}X^{2}Y-k_{3}BX-k_{4}X,$$

(13b) $$\frac{dY}{dt}=-k_{2}X^{2}Y+k_{3}BX,$$

(13c) A=0.5,B=3.

**Figure 16:**
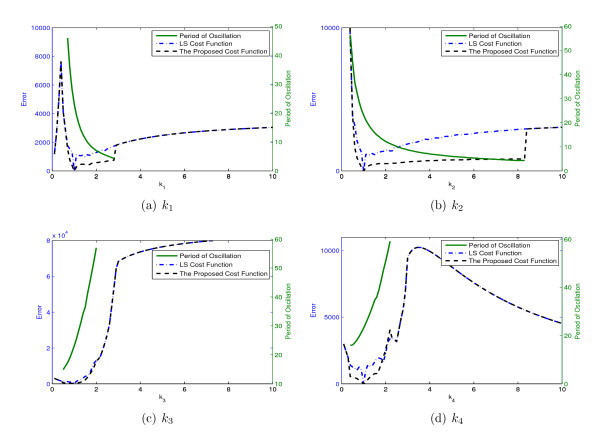
**The values of the LS cost functions, the proposed cost function and the fundamental period of the data for different values of 4 parameters of Brusselator model *k*_1_, *k*_2_, *k*_3_, and *k*_4_**.

Figure 16 shows the values of the two cost functions together with the fundamental period of the data (the green trajectories) for different values of four parameters of the system.

Figure 16a shows the fundamental period of sustained oscillation falls from 45.9 to 4.3 for *k*_1 _in the range [0.7 2.8]. This change in the fundamental period again produce irregularities in the LS cost function over this interval. The proposed cost function, on the other hand, maintains good surface properties in spite of this change in the fundamental period of the 22 system. This further verifies that the proposed cost function is able to address the irregularities of the LS cost function resulting from sustained dynamics embedded in the dynamics used to evaluate the cost function.

#### 3.3.1 Results of parameter estimation

**Figure 17:**
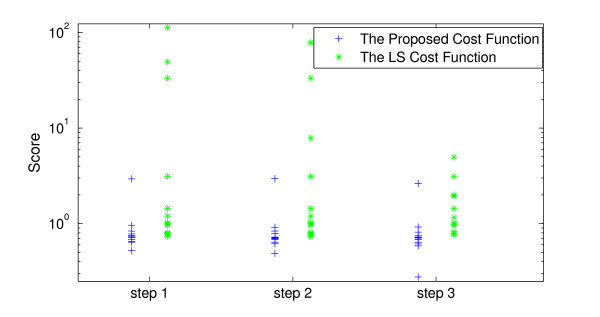
**The comparison of the computed scores resulted from the cost functions in 3 steps of optimization: Step 1: results of ga routine**. Step 2: results of fmincon routine. Step 3: results of fminsearch routine.

This section shows the results of 15 runs of optimization for the Brusselator model using 100 samples of only [Y] component with sampling rate = 1 (sample/hour) and AWGN noise with SNR = 20. We removed the noise using the proposed denoising approach. The RMSE between the noisy samples and their real values was 0.0971, which was suppressed to 0.0570 after denoising. The population size was set to 100 and the number of generations equals 50. The computed scores for the estimated parameters from the 15 runs of optimization are shown in Figure 17 and Table 4. We calculate $N_{T_{i}}=23$ for calculating the score of (11) for the Brusselator model. The results again demonstrate visually and numerically that the optimization routine performs better using the proposed cost function than the LS cost function in all steps even in presence of noise. These results are also consistent with our visual inspections of the cost functions in Figure 16.

**Table 3:** The results of optimization with minimum score for Brusselator model.

Parameter	Nominal value	Estimation of the proposed cost function	Estimation of the LS cost function
*k*_1_	1	0.9912	**1.4064**
*k*_2_	1	0.9112	**0.8492**
*k*_3_	1	0.9526	**0.8944**
*k*_4_	1	0.9335	**1.6732**
Score	0.3128	0.2763	0.7619

The values of kinetic parameters with minimum score derived from optimization using the proposed cost function and the LS cost function for the Brusselator model. The bold values are the ones that are estimated incorrectly (error more than 10%)

**Table 4:** Statistics of optimization results for Brusselator model.

	Step 1		Step 2		Step 3	
	
	Proposed	LS	Proposed	LS	Proposed	LS
Mean	0.8857	14.8167	0.8688	9.4746	0.8177	1.5517
Median	0.7336	1.0179	0.7116	1.0179	0.7107	1.0179
Min	0.5207	0.7323	0.4879	0.7323	0.2763	0.7619

Mean, median, and minimum of the score values shown in Figure 17

The derived results with the lowest score out of 15 runs for the LS cost function and the proposed cost function are in Table 3.

Table 3 shows that the resulting overall error for the proposed cost function is lower than that of the LS cost function. All four parameters were estimated incorrectly using the LS cost function, while they were estimated almost accurately using the proposed cost function.

### 3.4 Parameter estimation results for five-state Goldbeter model

The *D. melanogaster *circadian model of Goldbeter [27] was investigated in the third study. This model is also available in BioModels database [34] (BIOMD0000000016). Here, the circadian oscillations of PER is modeled with five states: PER mRNA [M], PER protein [P0], the mono-phosphorylated form [P1], the bi-phosphorylated form [P2] and nuclear PER [PN]. This five-state model has 18 kinetic parameters. The ODE model of the system is shown in 14. The nominal values of the 18 kinetic parameters of this system are available in Table 5.

(14a) $$\frac{dM}{dt}=v_{s}\frac{K_{I}^{n}}{K_{I}^{n}+P_{N}^{n}}-v_{m}\frac{M}{K_{m}+M}$$

(14b) $$\frac{dP_{0}}{dt}=k_{s}M-V_{1}\frac{P_{0}}{K_{1}+P_{0}}+V_{2}\frac{P_{1}}{K_{2}+P_{1}}$$

(14c) $$\frac{dP_{1}}{dt}=V_{1}\frac{P_{0}}{K_{1}+P_{0}}-V_{2}\frac{P_{1}}{K_{2}+P_{1}}-V_{3}\frac{P_{1}}{K_{3}+P_{1}}-V_{4}\frac{P_{2}}{K_{4}+P_{2}}$$

(14d) $$\frac{dP_{2}}{dt}=V_{3}\frac{p_{1}}{K_{3}+p_{1}}-V_{4}\frac{P_{2}}{K_{4}+P_{2}}-k_{1}P_{2}+k_{2}P_{N}-v_{d}\frac{P_{2}}{K_{d}+P_{2}}$$

(14e) $$\frac{dPN}{dt}=k_{1}P_{2}-k_{2}P_{N}$$

**Figure 18:**
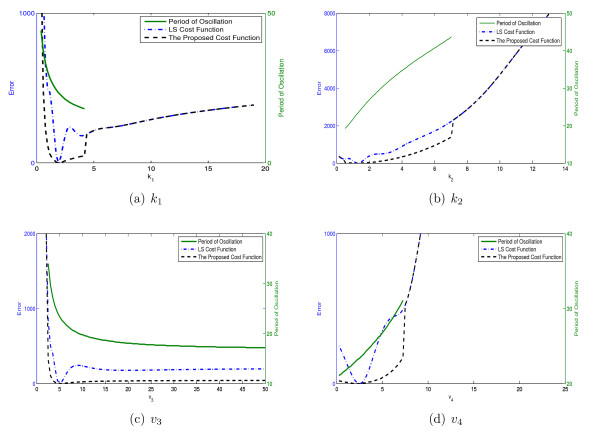
**The values of the LS cost functions, the proposed cost function and the fundamental period of the data for different values of 4 parameters of Goldbeter model *k*_1_, *k*_2_, *v*_3_, and *v*_4_**.

Figure 18 shows the values of the two cost function together with the fundamental period of the data (the green trajectories) along different values of four parameters of the system.

It could be seen in all figures that the changes in period of the oscillation does not produce significant irregularities in the LS cost function surface, which is different than previous examples. Figure 18b, for instance, shows the changes of period for *k*_2 _in the interval [0.4 2]. However, there are not multiple basins of attractions along the *k*_2 _direction in spite of these changes in fundamental period. This is due to the fact that the LS cost function changes over orders of magnitudes along this parameter direction in a way that the produced ripples has little effect on the monotonicity of the LS cost function. This extreme change in the LS cost function (approximately from 400 to 2200 for *k*_2 _over the interval [0.4 2]) happens because the peak to peak magnitude of the sustained oscillations of the simulated data also increases in order of magnitudes along this parameter direction. For example, the peak of the [*P*_2_] increases from 0.25 to 1.5 for *k*_2 _over the interval [0.4 2].

The proposed cost function still shows good surface characteristics although it was not much different than the already favorable characteristics of the LS cost function. Thus, it is expected that both of these cost functions would perform almost similar in the optimization process.

#### 3.4.1 Parameter estimation results

**Figure 19:**
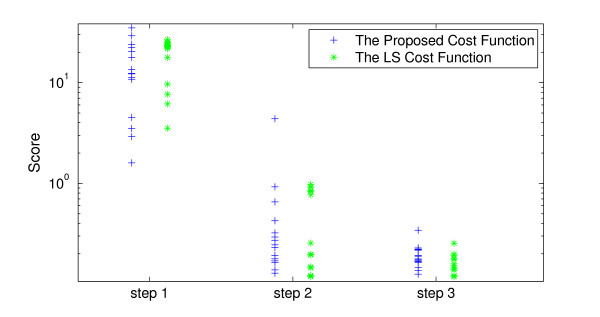
**The comparison of the computed scores resulted from the cost functions in 3 steps of optimization: Step 1: results of ga routine**. Step 2: results of fmincon routine. Step 3: Results of fminsearch routine.

This section shows the results of 15 optimization runs using 100 samples of [*M*], [*P*_0_], [*P*_1_], [*P*_2_], and [*P_N_*] components with the sampling rate = 1 (sample/hour) and AWGN noise with SNR = 20. We suppressed the noise using the proposed denoising approach. The RMSE between the noisy samples and their real values were 0.1012, which was suppressed to 0.04906 after denoising. We calculated $N_{T_{i}}=23$ for the score in (11). The results of 15 optimization runs are shown in Figure 19 and Table 6. This shows that the performances of the LS cost function and the proposed cost function are almost the same in all steps. These results are also consistent with our visual inspections of the cost functions in Figure 18.

**Table 5:** The Result of Optimization with Minimum Score for Goldbeter Model.

Parameter	Nominal value	Estimation of the proposed cost function	Estimation of the LS cost function
*v_s _*	0.76	0.6980	**0.6275**
*K_I _*	1	0.9996	0.9400
*n*	4	**4.9920**	**5.7232**
*v_m _*	0.65	0.5972	**0.5559**
*K_m _*	0.5	0.5056	**0.7412**
*k_s _*	0.38	0.3677	0.3732
*v*_1_	3.2	**3.8093**	3.1552
*K*_1_	2	**2.6488**	1.8288
*v*_2_	1.58	**3.1221**	**1.2836**
*K*_2_	2	**4.4760**	**1.1952**
*v*_3_	5	**8.8000**	4.5100
*K*_3_	2	**4.6696**	2.0120
*v*_4_	2.5	**5.6410**	**2.8120**
*K*_4_	2	**7.0128**	**3.0704**
*v_d _*	0.95	0.9713	0.9614
*K_d _*	0.2	**0.2413**	**0.2250**
*k*_1_	1.9	1.7541	**2.1944**

The values of kinetic parameters with minimum score derived from optimization using the proposed cost function and the LS cost function for the five-state Goldbeter model. The bold values are the ones that are estimated incorrectly (error more than 10%)

**Table 6:** Statistics of optimization results for Goldbeter model.

	Step 1		Step 2		Step 3	
	
	Proposed	LS	Proposed	LS	Proposed	LS
Mean	14.7428	18.8913	0.5812	0.4939	0.1914	0.1585
Median	12.3454	22.7683	0.2456	0.2553	0.1778	0.1446
Min	1.5970	3.5281	0.1282	0.1183	0.1255	0.1183

Mean, median and minimum of the score values shown in Figure 19

The derived results with the minimum score out of 15 runs for the LS cost function and the proposed cost function are shown in Table 5.

Table 5 shows that 8 out of 18 parameters were estimated within 10% of their nominal value for the proposed cost function as opposed to 7 out of 18 for the LS cost function. This shows a wide range of parameters have similar dynamics. This is due to system sloppiness that was also mentioned for the Tyson model. Our proposed cost function takes into account the accuracy of dynamics, which is similar to the LS cost function. Therefore, this may results in moderate accuracy in parameter values because of the sloppiness.

## 4 Conclusions

This article addresses the issue of kinetic parameter estimation in oscillatory biochemical systems. We showed that the LS cost function for oscillatory systems results in surface characteristics that potentially hinder the performance of optimization routines used to estimate kinetic parameters. Thus, we suggested a new cost function with more favorable surface properties which leads to improved results for parameter estimation. This cost function integrates temporal information with periodic information embedded in measurements used to estimate these parameters. This generalized cost function also needs less first principles knowledge to generate the cost function in comparison to the previous developed methods for oscillatory systems. We tested our cost function using three benchmark oscillatory biochemical pathways and compared our proposed objective function with the traditional LS cost function in several optimization runs using noisy measurements. The comparison of the results verified that the optimization performed more effectively using our 26 proposed cost function as compared to the traditional LS cost function. Furthermore, we introduced a wavelet hardthresholding approach for noise removal. This modified approach is able to suppress noise in oscillatory data better than the traditional wavelet thresholding approach. This, together with the proposed objective function will result in more accurate kinetic parameters that will eventually lead to biochemical models that are more precise, predictable and controllable. There are, however, unsolved issues with sloppiness of biochemical pathways [35,36], which require further investigation especially for oscillatory biochemical pathways.

## Competing interests

The authors declare that they have no competing interests.
